# 2-(3,4-Dimethyl-5,5-dioxo-2*H*,4*H*-pyrazolo­[4,3-*c*][1,2]benzothia­zin-2-yl)-*N*-(2-fluoro­benz­yl)acetamide

**DOI:** 10.1107/S160053681203187X

**Published:** 2012-07-18

**Authors:** Matloob Ahmad, Hamid Latif Siddiqui, Naveed Ahmad, Sana Aslam, Masood Parvez

**Affiliations:** a Department of Chemistry, Government College University, Faisalabad 38000, Pakistan; b Institute of Chemistry, University of the Punjab, Lahore 54590, Pakistan; c Department of Chemistry, The University of Calgary, 2500 University Drive NW, Calgary, Alberta, Canada T2N 1N4

## Abstract

In the title mol­ecule, C_20_H_19_FN_4_O_3_S, the heterocyclic thia­zine ring adopts a half-chair conformation with the S atom displaced by 0.668 (4) Å from the mean plane formed by the remaining ring atoms. The mean planes of the benzene and pyrazole rings are inclined with respect to each other at a dihedral angle of 17.4 (3)°. The acetamide chain (O/N/C/C/C) linking the pyrazole and 2-fluoro­benzyl rings is essentially planar (r.m.s. deviation = 0.030 Å) and forms dihedral angles with the mean planes of these rings of 78.8 (2) and 78.89 (14)°, respectively. The crystal structure is stabilized by N—H⋯O and C—H⋯O hydrogen-bonding inter­actions, resulting in a six-membered ring with an *R*_2_^1^(6) motif, while C—H⋯O and C—H⋯F hydrogen-bonding inter­actions result in chains of mol­ecules lying along the *c* axis in a zigzag fashion.

## Related literature

For biological activities of benzothia­zine derivatives, see: Turck *et al.* (1996 ▶); Silverstein *et al.* (2000 ▶); Lombardino *et al.* (1973 ▶); Zinnes *et al.* (1973 ▶); Ahmad, Siddiqui, Ahmad *et al.* (2010 ▶); Ahmad, Siddiqui, Zia-ur-Rehman & Parvez (2010 ▶). For related crystal structures, see: Siddiqui *et al.* (2008 ▶, 2009 ▶). For graph-set notations, see: Bernstein *et al.* (1995 ▶).
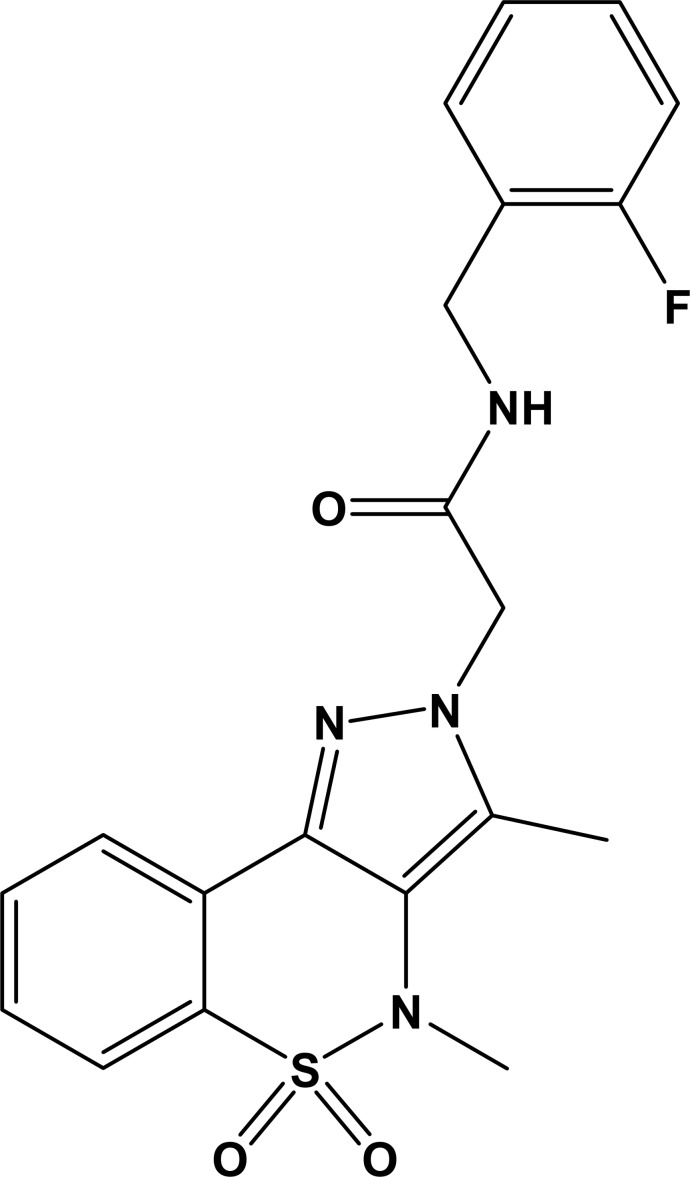


## Experimental

### 

#### Crystal data

C_20_H_19_FN_4_O_3_S
*M* *_r_* = 414.45
Orthorhombic, 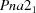

*a* = 27.4331 (15) Å
*b* = 7.4519 (5) Å
*c* = 9.2598 (6) Å
*V* = 1893.0 (2) Å^3^
*Z* = 4
Cu *K*α radiation
μ = 1.88 mm^−1^
*T* = 173 K
0.12 × 0.06 × 0.05 mm

#### Data collection

Bruker SMART APEXII CCD diffractometer
Absorption correction: multi-scan (*SADABS*; Bruker, 2004 ▶) *T*_min_ = 0.806, *T*_max_ = 0.912
17539 measured reflections
3009 independent reflections
2730 reflections with *I* > 2σ(*I*)
*R*_int_ = 0.029

#### Refinement

*R*[*F* ^2^ > 2σ(*F* ^2^)] = 0.049
*wR*(*F* ^2^) = 0.146
*S* = 1.09
3009 reflections
264 parameters
1 restraint
H-atom parameters constrained
Δρ_max_ = 1.05 e Å^−3^
Δρ_min_ = −0.45 e Å^−3^
Absolute structure: Flack (1983 ▶), 1207 Friedel pairs
Flack parameter: 0.05 (3)

### 

Data collection: *APEX2* (Bruker, 2004 ▶); cell refinement: *SAINT* (Bruker, 2004 ▶); data reduction: *SAINT* and *XPREP* (Bruker, 2004 ▶); program(s) used to solve structure: *SHELXS97* (Sheldrick, 2008 ▶); program(s) used to refine structure: *SHELXL97* (Sheldrick, 2008 ▶); molecular graphics: *ORTEP-3 for Windows* (Farrugia, 1997 ▶); software used to prepare material for publication: *SHELXL97*.

## Supplementary Material

Crystal structure: contains datablock(s) global, I. DOI: 10.1107/S160053681203187X/qm2076sup1.cif

Structure factors: contains datablock(s) I. DOI: 10.1107/S160053681203187X/qm2076Isup2.hkl

Supplementary material file. DOI: 10.1107/S160053681203187X/qm2076Isup3.cml

Additional supplementary materials:  crystallographic information; 3D view; checkCIF report

## Figures and Tables

**Table 1:** Hydrogen-bond geometry (Å, °)

*D*—H⋯*A*	*D*—H	H⋯*A*	*D*⋯*A*	*D*—H⋯*A*
N4—H4*A*⋯O3^i^	0.88	2.09	2.940 (5)	162
C3—H3⋯O2^ii^	0.95	2.50	3.366 (6)	152
C14—H14*A*⋯F1^iii^	0.99	2.53	3.202 (6)	125
C12—H12*B*⋯O3^i^	0.99	2.42	3.195 (6)	135
C14—H14*B*⋯F1	0.99	2.42	2.820 (6)	103

Symmetry codes: (i) 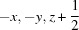; (ii) 
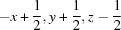
; (iii) 
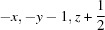
.

**Fig. 1.**
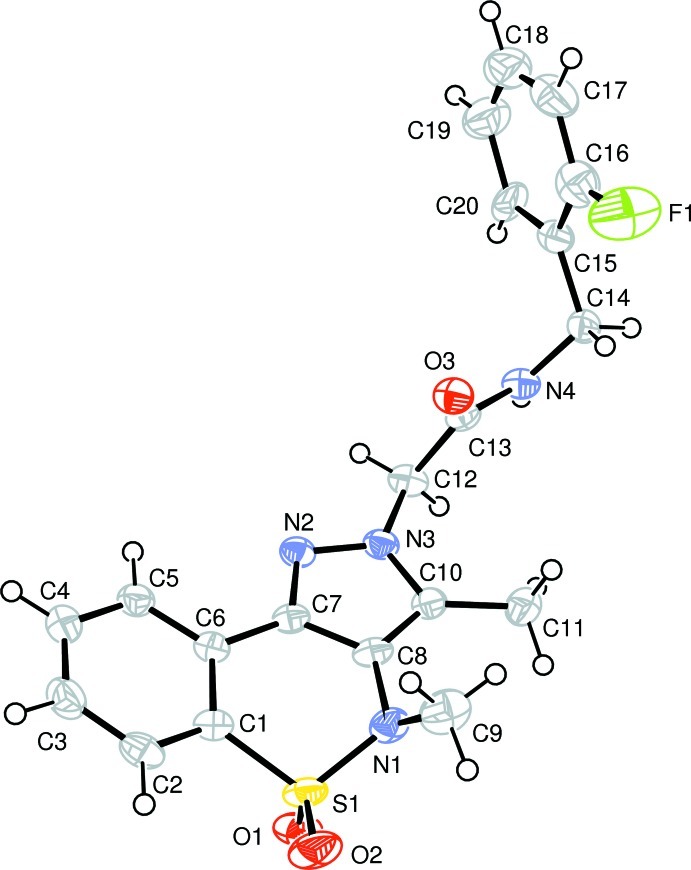
The molecular structure of the title compound with the atom numbering scheme. Displacement ellipsoids are drawn at the 50% probability level. H atoms are presented as small spheres of arbitrary radius.

**Fig. 2.**
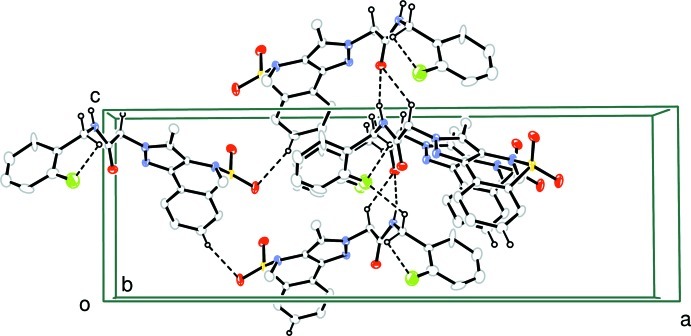
A part of the unit cell of the title compound showing hydrogen bonding interactions (dotted lines). H atoms non-participating in hydrogen-bonding were omitted for clarity.

## supplementary crystallographic information

### Comment

Oxicam drugs are benzothiazine based carboxamides which are well known for their potent anti-inflammatory and analgesic actions (Turck *et al.*, 1996; Lombardino *et al.*, 1973; Zinnes *et al.*, 1973). On the other hand, celecoxib, a pyrazole compound is an anti-inflammatory drug and a selective inhibitor of the cox-2 enzyme (Silverstein *et al.*, 2000). Keeping in view these features, we perceived that pyrazolobenzothiazine nucleus has a broad potential for biologically active molecules. We have prepared pyrazolobenzothiazines which are structural hybrids of both of these medicinally important heterocycles (Ahmad, Siddiqui, Ahmad, Parvez *et al.* (2010); Ahmad, Siddiqui, Zia-ur-Rehman & Parvez (2010)). In this article we report the crystal structure of the title molecule.

The bond distances and angles in the title compound (Fig. 1) agree very well with the corresponding bond distances and angles reported in closely related compounds (Siddiqui *et al.*, 2008; 2009). The heterocyclic thiazine ring adopts a half chair conformation with atom S1 displaced by 0.668 (4) Å, from the mean plane formed by the remaining ring atoms (r.m.s. deviation 0.036 Å for N1/C1/C6–C8 atoms). The mean-plane of the benzene ring C1–C6 makes a dihedral angle 17.4 (3)° with the mean-plane of the pyrazolyl ring (N2/N3/C7/C8/C10). The acetamide chain (O3/N4/C12–C14) linking the pyrazolyl and 2-fluorobenzyl rings is essentially planar (r.m.s. deviation 0.030 Å) and forms dihedral angles with the mean-planes of these rings 78.8 (2) and 78.89 (14)°, respectively.

The crystal structure is stabilized by intermolecular hydrogen bonding interactions (Fig. 2 and Table 1). The hydrogen bonds N4—H4A···O3 and C12—H12B···O3 result in a six membered rings in *R*_2_^1^(6) motif (Bernstein *et al.*, 1995) while C3—H3···O2 and C14—H14A···F1 hydrogen bonding interactions result in chains of molecules lying along the *c*-axis in a zigzag fashion.

### Experimental

3,4-Dimethyl-5,5-dioxidopyrazolo[4,3-*c*][1,2] benzothiazin-2(4*H*)-yl acetic acid (1.013 g, 3.3 mmol) was dissolved in toluene:THF (2:1) and boran-THF complex (1.1 mmol) was added. The reaction mixture was stirred for 40 minutes and 2-flourobenzyl amine (0.412 g, 3.3 mmol) added. The contents of the flask were refluxed for 5 h. The solvent was evaporated under vacuum and the product was purified by column chromatography. Colorless crystals were grown from an ethyl acetate solution which were used for X-ray crystallographic studies; m.p. 419–420 K.

### Refinement

All H atoms were positioned geometrically and refined using a riding model, with N—H = 0.88 Å and C—H = 0.95, 0.98 and 0.99 Å, for aryl, methyl and methylene H-atoms, respectively. The *U*_iso_(H) were allowed at 1.5*U*_eq_(methyl C) or 1.2*U*_eq_(the rest of the C/N). An absolute structure was determined by the Flack method (Flack, 1983) using 1207 Friedel pairs of reflections which were not merged.

### Crystal data

**Table:**

C_20_H_19_FN_4_O_3_S	*F*(000) = 864
*M**_r_* = 414.45	*D*_x_ = 1.454 Mg m^−^^3^
Orthorhombic, *P**n**a*2_1_	Cu *K*α radiation, λ = 1.54178 Å
Hall symbol: P 2c -2n	Cell parameters from 8539 reflections
*a* = 27.4331 (15) Å	θ = 3.2–67.4°
*b* = 7.4519 (5) Å	µ = 1.88 mm^−^^1^
*c* = 9.2598 (6) Å	*T* = 173 K
*V* = 1893.0 (2) Å^3^	Needle, colorless
*Z* = 4	0.12 × 0.06 × 0.05 mm

### Data collection

**Table:**

Bruker SMART APEXII CCD diffractometer	3009 independent reflections
Radiation source: fine-focus sealed tube	2730 reflections with *I* > 2σ(*I*)
Graphite monochromator	*R*_int_ = 0.029
ω and φ scans	θ_max_ = 68.1°, θ_min_ = 3.2°
Absorption correction: multi-scan (*SADABS*; Bruker, 2004)	*h* = −32→32
*T*_min_ = 0.806, *T*_max_ = 0.912	*k* = −8→8
17539 measured reflections	*l* = −10→8

### Refinement

**Table:**

Refinement on *F*^2^	Secondary atom site location: difference Fourier map
Least-squares matrix: full	Hydrogen site location: inferred from neighbouring sites
*R*[*F*^2^ > 2σ(*F*^2^)] = 0.049	H-atom parameters constrained
*wR*(*F*^2^) = 0.146	*w* = 1/[σ^2^(*F*_o_^2^) + (0.0785*P*)^2^ + 2.144*P*] where *P* = (*F*_o_^2^ + 2*F*_c_^2^)/3
*S* = 1.09	(Δ/σ)_max_ < 0.001
3009 reflections	Δρ_max_ = 1.05 e Å^−^^3^
264 parameters	Δρ_min_ = −0.45 e Å^−^^3^
1 restraint	Absolute structure: Flack (1983), 1207 Friedel pairs
Primary atom site location: structure-invariant direct methods	Flack parameter: 0.05 (3)

### Special details

**Table:**

Geometry. All e.s.d.'s (except the e.s.d. in the dihedral angle between two l.s. planes) are estimated using the full covariance matrix. The cell e.s.d.'s are taken into account individually in the estimation of e.s.d.'s in distances, angles and torsion angles; correlations between e.s.d.'s in cell parameters are only used when they are defined by crystal symmetry. An approximate (isotropic) treatment of cell e.s.d.'s is used for estimating e.s.d.'s involving l.s. planes.
Refinement. Refinement of *F*^2^ against ALL reflections. The weighted *R*-factor *wR* and goodness of fit *S* are based on *F*^2^, conventional *R*-factors *R* are based on *F*, with *F* set to zero for negative *F*^2^. The threshold expression of *F*^2^ > σ(*F*^2^) is used only for calculating *R*-factors(gt) *etc*. and is not relevant to the choice of reflections for refinement. *R*-factors based on *F*^2^ are statistically about twice as large as those based on *F*, and *R*- factors based on ALL data will be even larger.

### Fractional atomic coordinates and isotropic or equivalent isotropic displacement parameters (Å^2^)

**Table:**

	*x*	*y*	*z*	*U*_iso_*/*U*_eq_	
C1	0.18005 (14)	0.5585 (5)	0.5621 (5)	0.0298 (9)	
C2	0.19683 (14)	0.6653 (6)	0.4508 (5)	0.0366 (10)	
H2	0.2306	0.6695	0.4286	0.044*	
C3	0.16385 (16)	0.7660 (6)	0.3722 (6)	0.0410 (11)	
H3	0.1747	0.8363	0.2928	0.049*	
C4	0.11472 (16)	0.7642 (5)	0.4095 (6)	0.0411 (11)	
H4	0.0925	0.8391	0.3589	0.049*	
C5	0.09785 (14)	0.6551 (5)	0.5191 (5)	0.0330 (10)	
H5	0.0642	0.6552	0.5433	0.040*	
C6	0.12980 (13)	0.5455 (5)	0.5939 (5)	0.0276 (8)	
C7	0.11436 (12)	0.4057 (5)	0.6914 (5)	0.0279 (8)	
C8	0.14419 (13)	0.2643 (5)	0.7402 (5)	0.0294 (9)	
C9	0.21028 (17)	0.0897 (7)	0.6235 (6)	0.0487 (13)	
H9A	0.1937	−0.0172	0.6608	0.073*	
H9B	0.2456	0.0742	0.6326	0.073*	
H9C	0.2018	0.1066	0.5216	0.073*	
C10	0.11529 (13)	0.1512 (5)	0.8186 (5)	0.0309 (9)	
C11	0.12523 (17)	−0.0203 (6)	0.8981 (6)	0.0402 (11)	
H11A	0.1037	−0.0283	0.9824	0.060*	
H11B	0.1593	−0.0221	0.9301	0.060*	
H11C	0.1192	−0.1225	0.8340	0.060*	
C12	0.02597 (14)	0.1619 (5)	0.8796 (5)	0.0336 (9)	
H12A	−0.0003	0.2517	0.8664	0.040*	
H12B	0.0315	0.1467	0.9845	0.040*	
C13	0.00996 (13)	−0.0174 (5)	0.8142 (5)	0.0265 (8)	
C14	−0.02579 (14)	−0.3102 (5)	0.8631 (5)	0.0317 (9)	
H14A	−0.0295	−0.3838	0.9515	0.038*	
H14B	0.0014	−0.3620	0.8063	0.038*	
C15	−0.07211 (15)	−0.3255 (5)	0.7752 (5)	0.0321 (9)	
C16	−0.07666 (19)	−0.4367 (6)	0.6583 (6)	0.0504 (13)	
C17	−0.1212 (2)	−0.4580 (7)	0.5805 (6)	0.0593 (16)	
H17	−0.1236	−0.5374	0.5006	0.071*	
C18	−0.1600 (2)	−0.3605 (8)	0.6252 (8)	0.0618 (16)	
H18	−0.1898	−0.3701	0.5735	0.074*	
C19	−0.15798 (19)	−0.2509 (9)	0.7397 (8)	0.0635 (16)	
H19	−0.1864	−0.1850	0.7649	0.076*	
C20	−0.11537 (14)	−0.2278 (8)	0.8259 (9)	0.077 (2)	
H20	−0.1150	−0.1547	0.9099	0.093*	
F1	−0.03977 (13)	−0.5300 (6)	0.6187 (5)	0.0875 (13)	
N1	0.19490 (11)	0.2479 (4)	0.7068 (4)	0.0341 (9)	
N2	0.06857 (10)	0.3820 (4)	0.7375 (4)	0.0281 (7)	
N3	0.07015 (11)	0.2262 (4)	0.8123 (4)	0.0294 (7)	
N4	−0.01247 (11)	−0.1285 (5)	0.9057 (4)	0.0304 (8)	
H4A	−0.0193	−0.0912	0.9936	0.036*	
O1	0.22202 (11)	0.5389 (4)	0.8089 (4)	0.0455 (8)	
O2	0.26539 (10)	0.4115 (5)	0.5993 (4)	0.0522 (9)	
O3	0.01791 (9)	−0.0534 (4)	0.6870 (4)	0.0345 (6)	
S1	0.22083 (3)	0.44366 (14)	0.67509 (13)	0.0366 (3)	

### Atomic displacement parameters (Å^2^)

**Table:**

	*U*^11^	*U*^22^	*U*^33^	*U*^12^	*U*^13^	*U*^23^
C1	0.0245 (19)	0.035 (2)	0.030 (3)	−0.0034 (15)	−0.0003 (17)	−0.0045 (17)
C2	0.0277 (19)	0.041 (2)	0.041 (3)	−0.0089 (16)	0.0123 (19)	−0.003 (2)
C3	0.045 (2)	0.039 (2)	0.038 (3)	−0.0113 (19)	0.003 (2)	0.008 (2)
C4	0.037 (2)	0.032 (2)	0.055 (3)	−0.0024 (17)	−0.004 (2)	0.007 (2)
C5	0.0247 (18)	0.0297 (19)	0.044 (3)	−0.0011 (15)	−0.0004 (18)	−0.0020 (19)
C6	0.0256 (18)	0.0299 (18)	0.027 (2)	−0.0046 (15)	0.0001 (17)	−0.0017 (17)
C7	0.0202 (15)	0.0357 (19)	0.028 (2)	−0.0012 (13)	0.0007 (17)	−0.0018 (19)
C8	0.0192 (16)	0.041 (2)	0.028 (2)	−0.0003 (15)	−0.0001 (17)	−0.0041 (17)
C9	0.038 (2)	0.057 (3)	0.051 (3)	0.011 (2)	0.004 (2)	−0.014 (2)
C10	0.0257 (18)	0.0307 (19)	0.036 (3)	0.0002 (14)	−0.0043 (17)	−0.0015 (18)
C11	0.038 (2)	0.042 (2)	0.041 (3)	0.0007 (18)	−0.006 (2)	0.010 (2)
C12	0.0290 (19)	0.038 (2)	0.033 (3)	−0.0057 (16)	0.0026 (18)	−0.0058 (19)
C13	0.0202 (17)	0.036 (2)	0.023 (2)	0.0010 (14)	0.0011 (16)	0.0009 (18)
C14	0.0278 (18)	0.0296 (19)	0.038 (3)	−0.0008 (15)	0.0007 (18)	0.0023 (18)
C15	0.037 (2)	0.035 (2)	0.024 (2)	−0.0099 (16)	−0.0015 (17)	0.0037 (17)
C16	0.058 (3)	0.050 (3)	0.044 (3)	−0.003 (2)	0.004 (3)	0.003 (2)
C17	0.092 (4)	0.056 (3)	0.030 (3)	−0.029 (3)	−0.018 (3)	0.006 (2)
C18	0.047 (3)	0.070 (4)	0.069 (4)	−0.018 (3)	−0.008 (3)	0.022 (3)
C19	0.043 (3)	0.074 (4)	0.073 (5)	−0.004 (3)	−0.011 (3)	0.015 (4)
C20	0.019 (2)	0.072 (3)	0.141 (7)	−0.013 (2)	−0.021 (3)	0.068 (4)
F1	0.063 (2)	0.117 (3)	0.082 (3)	0.011 (2)	−0.0018 (19)	−0.036 (3)
N1	0.0213 (14)	0.0416 (18)	0.039 (2)	0.0041 (12)	0.0043 (15)	0.0026 (17)
N2	0.0225 (14)	0.0303 (17)	0.032 (2)	−0.0024 (12)	0.0031 (14)	−0.0018 (14)
N3	0.0235 (15)	0.0333 (16)	0.032 (2)	−0.0056 (12)	0.0006 (14)	0.0017 (15)
N4	0.0254 (15)	0.0374 (17)	0.028 (2)	−0.0041 (14)	0.0007 (14)	−0.0031 (15)
O1	0.0400 (17)	0.059 (2)	0.038 (2)	−0.0167 (13)	−0.0096 (15)	−0.0051 (17)
O2	0.0200 (13)	0.077 (2)	0.060 (2)	0.0022 (14)	0.0048 (15)	0.007 (2)
O3	0.0364 (14)	0.0427 (15)	0.0243 (17)	−0.0059 (11)	−0.0004 (14)	0.0020 (13)
S1	0.0184 (4)	0.0516 (6)	0.0399 (7)	−0.0042 (4)	−0.0017 (4)	0.0012 (5)

### Geometric parameters (Å, º)

**Table:**

C1—C2	1.381 (6)	C12—C13	1.531 (6)
C1—C6	1.413 (5)	C12—H12A	0.9900
C1—S1	1.755 (4)	C12—H12B	0.9900
C2—C3	1.383 (7)	C13—O3	1.227 (5)
C2—H2	0.9500	C13—N4	1.335 (5)
C3—C4	1.391 (6)	C14—N4	1.457 (5)
C3—H3	0.9500	C14—C15	1.514 (6)
C4—C5	1.380 (7)	C14—H14A	0.9900
C4—H4	0.9500	C14—H14B	0.9900
C5—C6	1.384 (6)	C15—C16	1.369 (7)
C5—H5	0.9500	C15—C20	1.469 (7)
C6—C7	1.443 (6)	C16—F1	1.282 (6)
C7—N2	1.339 (5)	C16—C17	1.428 (8)
C7—C8	1.408 (5)	C17—C18	1.354 (9)
C8—C10	1.366 (6)	C17—H17	0.9500
C8—N1	1.430 (5)	C18—C19	1.340 (9)
C9—N1	1.471 (6)	C18—H18	0.9500
C9—H9A	0.9800	C19—C20	1.425 (8)
C9—H9B	0.9800	C19—H19	0.9500
C9—H9C	0.9800	C20—H20	0.9500
C10—N3	1.360 (5)	N1—S1	1.650 (3)
C10—C11	1.500 (6)	N2—N3	1.353 (5)
C11—H11A	0.9800	N4—H4A	0.8800
C11—H11B	0.9800	O1—S1	1.429 (4)
C11—H11C	0.9800	O2—S1	1.430 (3)
C12—N3	1.444 (5)		
			
C2—C1—C6	121.3 (4)	H12A—C12—H12B	108.0
C2—C1—S1	120.9 (3)	O3—C13—N4	123.7 (4)
C6—C1—S1	117.7 (3)	O3—C13—C12	121.3 (4)
C1—C2—C3	119.2 (4)	N4—C13—C12	115.0 (4)
C1—C2—H2	120.4	N4—C14—C15	115.2 (3)
C3—C2—H2	120.4	N4—C14—H14A	108.5
C2—C3—C4	119.8 (4)	C15—C14—H14A	108.5
C2—C3—H3	120.1	N4—C14—H14B	108.5
C4—C3—H3	120.1	C15—C14—H14B	108.5
C5—C4—C3	120.9 (4)	H14A—C14—H14B	107.5
C5—C4—H4	119.6	C16—C15—C20	118.6 (5)
C3—C4—H4	119.6	C16—C15—C14	123.2 (4)
C4—C5—C6	120.2 (4)	C20—C15—C14	118.0 (4)
C4—C5—H5	119.9	F1—C16—C15	118.9 (5)
C6—C5—H5	119.9	F1—C16—C17	118.1 (5)
C5—C6—C1	118.3 (4)	C15—C16—C17	123.0 (5)
C5—C6—C7	123.6 (3)	C18—C17—C16	117.4 (5)
C1—C6—C7	117.8 (3)	C18—C17—H17	121.3
N2—C7—C8	110.2 (3)	C16—C17—H17	121.3
N2—C7—C6	124.8 (3)	C19—C18—C17	122.4 (6)
C8—C7—C6	124.8 (3)	C19—C18—H18	118.8
C10—C8—C7	107.1 (3)	C17—C18—H18	118.8
C10—C8—N1	128.8 (4)	C18—C19—C20	123.4 (6)
C7—C8—N1	124.1 (4)	C18—C19—H19	118.3
N1—C9—H9A	109.5	C20—C19—H19	118.3
N1—C9—H9B	109.5	C19—C20—C15	115.1 (7)
H9A—C9—H9B	109.5	C19—C20—H20	122.5
N1—C9—H9C	109.5	C15—C20—H20	122.5
H9A—C9—H9C	109.5	C8—N1—C9	117.5 (3)
H9B—C9—H9C	109.5	C8—N1—S1	112.4 (3)
N3—C10—C8	104.6 (3)	C9—N1—S1	119.5 (3)
N3—C10—C11	122.4 (4)	C7—N2—N3	104.3 (3)
C8—C10—C11	132.9 (4)	N2—N3—C10	113.8 (3)
C10—C11—H11A	109.5	N2—N3—C12	118.6 (3)
C10—C11—H11B	109.5	C10—N3—C12	127.6 (3)
H11A—C11—H11B	109.5	C13—N4—C14	121.3 (4)
C10—C11—H11C	109.5	C13—N4—H4A	119.3
H11A—C11—H11C	109.5	C14—N4—H4A	119.3
H11B—C11—H11C	109.5	O1—S1—O2	119.3 (2)
N3—C12—C13	111.1 (3)	O1—S1—N1	107.15 (19)
N3—C12—H12A	109.4	O2—S1—N1	107.9 (2)
C13—C12—H12A	109.4	O1—S1—C1	106.9 (2)
N3—C12—H12B	109.4	O2—S1—C1	109.5 (2)
C13—C12—H12B	109.4	N1—S1—C1	105.23 (17)
			
C6—C1—C2—C3	2.2 (7)	C17—C18—C19—C20	−0.7 (9)
S1—C1—C2—C3	−174.3 (3)	C18—C19—C20—C15	3.6 (8)
C1—C2—C3—C4	2.6 (7)	C16—C15—C20—C19	−4.1 (7)
C2—C3—C4—C5	−3.7 (7)	C14—C15—C20—C19	−178.8 (4)
C3—C4—C5—C6	0.1 (7)	C10—C8—N1—C9	60.7 (6)
C4—C5—C6—C1	4.5 (6)	C7—C8—N1—C9	−117.5 (5)
C4—C5—C6—C7	−168.7 (4)	C10—C8—N1—S1	−154.7 (4)
C2—C1—C6—C5	−5.7 (6)	C7—C8—N1—S1	27.0 (5)
S1—C1—C6—C5	170.9 (3)	C8—C7—N2—N3	−0.8 (5)
C2—C1—C6—C7	167.9 (4)	C6—C7—N2—N3	173.4 (4)
S1—C1—C6—C7	−15.5 (5)	C7—N2—N3—C10	1.3 (5)
C5—C6—C7—N2	−10.3 (7)	C7—N2—N3—C12	179.1 (4)
C1—C6—C7—N2	176.5 (4)	C8—C10—N3—N2	−1.3 (5)
C5—C6—C7—C8	163.0 (4)	C11—C10—N3—N2	179.0 (4)
C1—C6—C7—C8	−10.2 (6)	C8—C10—N3—C12	−178.9 (4)
N2—C7—C8—C10	0.0 (5)	C11—C10—N3—C12	1.5 (7)
C6—C7—C8—C10	−174.1 (4)	C13—C12—N3—N2	116.5 (4)
N2—C7—C8—N1	178.6 (4)	C13—C12—N3—C10	−66.1 (6)
C6—C7—C8—N1	4.5 (7)	O3—C13—N4—C14	5.2 (6)
C7—C8—C10—N3	0.8 (5)	C12—C13—N4—C14	−174.2 (3)
N1—C8—C10—N3	−177.8 (4)	C15—C14—N4—C13	−80.3 (5)
C7—C8—C10—C11	−179.7 (5)	C8—N1—S1—O1	68.7 (3)
N1—C8—C10—C11	1.8 (8)	C9—N1—S1—O1	−147.5 (4)
N3—C12—C13—O3	−31.9 (5)	C8—N1—S1—O2	−161.6 (3)
N3—C12—C13—N4	147.6 (3)	C9—N1—S1—O2	−17.8 (4)
N4—C14—C15—C16	138.4 (4)	C8—N1—S1—C1	−44.7 (3)
N4—C14—C15—C20	−47.1 (5)	C9—N1—S1—C1	99.0 (4)
C20—C15—C16—F1	−176.0 (5)	C2—C1—S1—O1	104.4 (4)
C14—C15—C16—F1	−1.6 (7)	C6—C1—S1—O1	−72.2 (3)
C20—C15—C16—C17	2.0 (7)	C2—C1—S1—O2	−26.1 (4)
C14—C15—C16—C17	176.4 (4)	C6—C1—S1—O2	157.2 (3)
F1—C16—C17—C18	179.0 (5)	C2—C1—S1—N1	−141.9 (4)
C15—C16—C17—C18	0.9 (8)	C6—C1—S1—N1	41.5 (4)
C16—C17—C18—C19	−1.6 (8)		

### Hydrogen-bond geometry (Å, º)

**Table:**

*D*—H···*A*	*D*—H	H···*A*	*D*···*A*	*D*—H···*A*
N4—H4*A*···O3^i^	0.88	2.09	2.940 (5)	162
C3—H3···O2^ii^	0.95	2.50	3.366 (6)	152
C14—H14*A*···F1^iii^	0.99	2.53	3.202 (6)	125
C12—H12*B*···O3^i^	0.99	2.42	3.195 (6)	135
C14—H14*B*···F1	0.99	2.42	2.820 (6)	103

Symmetry codes: (i) −*x*, −*y*, *z*+1/2; (ii) −*x*+1/2, *y*+1/2, *z*−1/2; (iii) −*x*, −*y*−1, *z*+1/2.
